# Ovarian Reserve Disorders, Can We Prevent Them? A Review

**DOI:** 10.3390/ijms232315426

**Published:** 2022-12-06

**Authors:** Limor Man, Nicole Lustgarten Guahmich, Nina Vyas, Shelun Tsai, Laury Arazi, Debra Lilienthal, Glenn Schattman, Zev Rosenwaks, Daylon James

**Affiliations:** Ronald O. Perelman and Claudia Cohen Center for Reproductive Medicine and Infertility, Weill Cornell Medicine, New York, NY 10007, USA

**Keywords:** ovarian reserve disorder, premature ovarian insufficiency, prevention, fertility preservation

## Abstract

The ovarian reserve is finite and begins declining from its peak at mid-gestation until only residual follicles remain as women approach menopause. Reduced ovarian reserve, or its extreme form, premature ovarian insufficiency, stems from multiple factors, including developmental, genetic, environmental exposures, autoimmune disease, or medical/surgical treatment. In many cases, the cause remains unknown and resulting infertility is not ultimately addressed by assisted reproductive technologies. Deciphering the mechanisms that underlie disorders of ovarian reserve could improve the outcomes for patients struggling with infertility, but these disorders are diverse and can be categorized in multiple ways. In this review, we will explore the topic from a perspective that emphasizes the prevention or mitigation of ovarian damage. The most desirable mode of fertoprotection is primary prevention (intervening before ablative influence occurs), as identifying toxic influences and deciphering the mechanisms by which they exert their effect can reduce or eliminate exposure and damage. Secondary prevention in the form of screening is not recommended broadly. Nevertheless, in some instances where a known genetic background exists in discrete families, screening is advised. As part of prenatal care, screening panels include some genetic diseases that can lead to infertility or subfertility. In these patients, early diagnosis could enable fertility preservation or changes in family-building plans. Finally, Tertiary Prevention (managing disease post-diagnosis) is critical. Reduced ovarian reserve has a major influence on physiology beyond fertility, including delayed/absent puberty or premature menopause. In these instances, proper diagnosis and medical therapy can reduce adverse effects. Here, we elaborate on these modes of prevention as well as proposed mechanisms that underlie ovarian reserve disorders.

## 1. Introduction

The term ovarian reserve describes a woman’s fertility potential when no other reproductive tract abnormalities are detected, based on the number and quality of eggs. The eggs reside in the ovary as part of a unique complex of cells called primordial follicles (PrF). Primordial follicles assemble in the ovary before birth, and their number peaks at mid-gestation. During the female’s reproductive life span, most follicles will undergo activation and atresia and once the ovarian reserve is mostly depleted, amenorrhea follows [1]. For 95% of women by 30 years old, only 12% of their maximum pre-birth PrF population is present, and by the age of 40 years, only 3% remains. Even across women of the same age, the number of PrFs within the ovarian reserve lies within a spectrum ranging from normal, diminished ovarian reserve (DOR), and premature ovarian insufficiency (POI). DOR is an intermediate state where the ovarian reserve is not at its normal reproductive potential, and fertility is compromised. The diagnosis of DOR is not straightforward, as most women have no signs or symptoms, but it is relatively common among women seeking care due to infertility. Common signs of DOR are the shortening of the menstrual cycle and lower estrogen levels that may induce symptoms like hot flashes, vaginal dryness, etc. The extreme form of DOR is POI, occurring at a frequency of between 1–3.7% [2,3,4] below the age of 40 and 0.1% below the age of 30 [2].

Ovarian reserve is evaluated by measuring antral follicular count (AFC) with ultrasound or measuring the serum levels of anti-Mullerian hormone (AMH). The European Society of Human Reproduction and Embryology (ESHRE) published guidelines for diagnosing primary and secondary POI [3]. The criteria are: 1. Women younger than 40 years at diagnosis; 2. at least four months of cycle irregularities (oligo/amenorrhea); and 3. elevated levels of Follicle-stimulating hormone (FSH) on two occasions at least a month apart. High FSH levels are necessary for POI diagnosis, but the threshold level is inconsistent. Goldenberg et al. [4] showed that, in women with primary amenorrhea, no follicles were found in ovarian biopsies when FSH serum levels were higher than 33 mIU/mL. In women with secondary amenorrhea, no ovarian follicles were found when the FSH was >40 mIU/mL. Remarkably, women with autoantibodies may be diagnosed with POI with lower FSH levels (>25 mIU/mL) and normal AMH levels [5].

It is imperative to distinguish between DOR and POI since women with POI face challenges beyond infertility and need appropriate care. Osteoporosis and cardiovascular morbidity are serious health concerns that affect women with POI [6]. Strikingly, for women who had prophylactic bilateral oophorectomy before 45, mortality was significantly higher compared to matched controls, mainly in women who did not receive estradiol until age 45 [7]. POI is also linked to mental disorders; like anxiety and depression [8]. Importantly, using hormonal therapy: oral contraception, ovarian hyperstimulation, or hormonal replacement therapy (HRT) does not cause POI but rather masks the condition. Some women can still conceive after being diagnosed with POI since ovarian activity may occur, especially early in the natural history of POI. There are reports that estimate a 5–12% chance of natural conception after POI diagnosis [9,10,11]. If a patient does not wish to conceive, contraception should be advised. In cases where POI has a genetic background that might affect the pregnancy and offspring, it should be discussed with the patient.

Most POI patients (about 86%) have secondary amenorrhea with a rapid decline in ovarian function and cessation of menstruation 12–24 months after the beginning of menstrual irregularity. Patients presenting with primary amenorrhea are more likely to have chromosomal aberrations [12], accounting for 10–20% of cases [13,14]. Genetic defects play a role in POI as well. Multiple genetic mutations in autosomal and sex chromosomes, as well as; mitochondrial genes, have been associated with this condition. Autoimmune disorders are more frequent in patients with POI than in the general population, and vice versa, especially certain autoimmune disorders [15], its prevalence in POI patients is about 12% [12]. Medical/surgical procedures also have a substantial role in the etiology of POI-iatrogenic cases are responsible for about 7% [12]. Finally, developmental defects and environmental influences can contribute to POI, yet even accounting for all these etiologies, some POI cases remain unexplained.

When translating basic science understandings into the medical field, we cannot stress enough the importance of understanding the underlying mechanisms. Upon understanding the mechanisms, we would aspire to prevent or at least attenuate the progression of the disease and minimize its ramifications. Unfortunately, it is not always possible, but there are some actionable strategies we will emphasize in this review. What can we do to serve patients and their family members better?

In this review manuscript, we will elaborate on: (1) Developmental abnormalities; (2) genetic background, including X chromosome defects and gene mutations (Turner syndrome, 47, XXX, fragile X syndrome), single gene defects causing POI, idiopathic; (3) infections: HIV, SAR-CoV-2 virus; (4) autoimmune disease; (5) medical treatment: Chemotherapy, radiation, and surgeries/procedures; and (6) environmental exposure. At the conclusion of each topic, we will introduce prevention strategies that are available in the current standard of clinical care, as well as some experimental or hypothetical approaches that may be available in the future. At the end of the manuscript, one can find Table 1 summarizing the available prevention approaches.

## 2. Developmental Abnormalities

The proper assembly and development of the human ovary depends on complex events that occur in synchrony, starting in utero and continuing after birth. First, PrFs, which comprise the ovarian reserve, are assembled and over the reproductive lifespan of a woman there are continuous waves of recruitment, growth, development, and maturation, culminating in ovulation of an oocyte competent to be fertilized. Our understanding of the formation and assembly of the ovary and its reserve stem from mammalian models, mainly murine. Here, we will bring a concise preview and highlight only key elements.

Early in embryogenesis post-fertilization and the creation of the zygote, multiple extrinsic factors contribute to the development of primordial germ cells (PGC). During early discrimination of PGC precursors from the somatic cells of the embryo [16], members of the bone morphogenetic protein (BMP) group of transforming growth factor beta (TGFb) superfamily [17] are necessary. BMP4 and BMP8B, secreted from the extraembryonic ectoderm [18], and BMP2, secreted from the visceral endoderm [19], are required. Once PGCs are allocated, they proliferate and migrate to the genital ridge, guided by chemoattractants. By seven weeks of gestation, the gonad remains bipotent, with the first inductive difference between an XX and XY germ cells in the genital ridge being the reactivation of the X chromosome in female PGCs. Soon after, the fate of these PGCs changes [16], and PGCs are directed along either male or female gametogenic pathways.

Steroidogenic factor 1 (SF1), a key determinant of female gonad identity, is regulated by Wilms tumor 1 (WT1), Lim homeobox 9 (LHX9), and chromobox homolog protein 2 (CBX2). The ovarian differentiation pathway is driven by R-Spondin1 (RSPO1), which increases the signaling of Wnt family member 4 (WNT4), and up-regulates b-catenin. B-catenin causes increased expression of WNT4 and other proteins, such as follistatin (FST), and antagonizes the testis pathway by destabilizing SOX9. Germ cells then continue to divide and enter meiosis in a process regulated by the key genes deleted in azoospermia-like (DAZL) and synaptonemal complex protein 3 (SYCP3). Retinoid acid is pivotal in meiosis during embryogenesis. The mesonephra near to the developing gonad express aldehyde dehydrogenases, enzymes that convert retinaldehyde to all-trans-retinoic acid (RA). RA induces STRA8 and SYCP3, which is translated in the presence of DAZL and becomes chromosomally localized as the germ cell becomes an oocyte and enters meiosis [16]. Germ cells are organized in clusters (nests) and lack surrounding somatic cells. When the germ cell cluster breaks down at 16 weeks of gestation, individual oocytes become surrounded by squamous pre-granulosa (flattened granulosa) cells to form PrFs. At this stage, most oocytes will undergo apoptosis [20]. By 16 to 20 weeks gestation, the fetal ovaries contain six to seven million oogonia [21]. The oocytes are arrested at the diplotene stage of meiosis I, and do not resume meiosis until postnatally, during ovarian folliculogenesis and ovulation. This population of PrFs serves as a resting and finite pool of oocytes available during the female reproductive life span. In the second half of gestation, there is substantial oocyte atresia leaving, between 500,000 and 2 million oocytes at birth [22], and by the onset of puberty the number of oocytes is decreased to between 300,000 and 500,000 [23]. Follicular mobilization and atresia then continue throughout adulthood until menopause, when only about 1000 PrFs remain in the ovaries.

Upon recruitment into the growing pool and throughout folliculogenesis, the oocyte increases in size. Meiotic resumption occurs following the surge in luteinizing hormone (LH), and the first polar body is released. At this point, the oocyte is arrested at MII, and ovulation results in its release into the fallopian tube. Fertilization with a spermatozoa leads to the completion of meiosis and the release of the second polar body [16]. These processes are highly coordinated and susceptible to disruption by genetic mutations, environmental exposures, and underlying medical illnesses at various points in ovarian development, potentially leading to abnormalities that could affect the ovarian reserve and lead to POI.

Numerous factors can disrupt normal developmental processes and either alter the ovarian structure and function or accelerate the depletion of oocytes. Gonadal dysgenesis, impaired development of the gonads, is a group of conditions where the gonads are underdeveloped and have a complete or partial loss of function. Most cases of ovarian dysgenesis are due to abnormalities in the X chromosome [24]. Individuals with 46, XX gonadal dysgenesis may present with primary amenorrhea, hypergonadotropic hypogonadism, and infertility. The uterus, fallopian tubes, upper vagina, and external genitalia are phenotypically normal in these individuals; however, they do not experience normal puberty. The ovarian tissue develops abnormally and is replaced by fibrous tissue resulting in characteristic “streak ovaries” [25]. In people with 46, XX, the etiology could be a genetic mutation or environmental factor(s) affecting normal ovarian development. Chemical exposures during fetal life have also been implicated in ovarian dysgenesis. Nonetheless, most of the supporting data is derived from animal studies due to the limited ability to obtain data on intrauterine exposure and correlate it with adverse outcomes in adulthood. One category of chemicals that have been implicated with ovarian dysgenesis if exposed in utero are endocrine disruptors, which can interfere with endogenous estrogens. For example, tamoxifen, a selective estrogen receptor modulator used for breast cancer treatment, has been shown to interfere with gonadal sex differentiation in mice [26]. Another category of medication exposure is analgesics. Acetaminophen was suggested to reduce the proliferation of germ cells and delay meiotic progression in mice and rats [27,28]. While these animal studies shed light on the impact of chemical exposures in utero and their potential impact on adult life, it is important to keep in mind that robust human epidemiological data is limited and often difficult to interpret.

*A. Primary Prevention*

Limiting and ideally avoiding chemical exposures as much as possible during pregnancy is preferable, with a particular emphasis on the avoidance of endocrine disruptors.

*B. Secondary Prevention*

For people with a family history of genetic abnormalities, developmental abnormalities, or POI, a genetic screening accompanied by proper genetic counseling, can potentially identify heritable mutations before conception. Prenatal diagnosis during pregnancy with chorionic villus sampling (CVS) or amniocentesis might also be considered.

*C. Tertiary Prevention*

Oocyte cryopreservation in early adulthood or teen years is a valid strategy. Oocyte cryopreservation warrants counseling with a reproductive endocrinologist (REI). Treatment with controlled ovarian hyperstimulation (COH), followed by oocyte retrieval and cryopreservation. This enables the patient to have a personal repository of eggs in the event that their ovarian reserve deteriorates at an accelerated pace, and they are unable to conceive naturally.

## 3. Genetic Background

Chromosomal etiologies are responsible for about 10–20% of cases of POI. The majority are structural abnormalities or aneuploidy of the X chromosome [13,14]. Nonetheless, it is plausible that a portion of idiopathic POI can also be traced to genetic causes. An abnormal karyotype is more frequent in women with primary amenorrhea (21%), as compared to secondary amenorrhea (11%) [13].

### 3.1. X Chromosome Defects and Gene Mutations

In general, X chromosome-linked defects contribute to most genetic cases with POI. Turner syndrome and its related defects are estimated to be at the frequency of 4–5% of POI, 47, XXX syndrome, 1–4%, and fragile X syndrome (*FMR1* premutation) 3–15% [29].

#### 3.1.1. Turner Syndrome

Turner syndrome affects 1 in 2000 to 1 in 2500 female births, making it the most common female sex chromosome anomaly and most common chromosomal defect in humans [30]. Additionally, as mentioned above, it is one of the most common causes of POI. Turner syndrome arises in cases of monosomy of the X chromosome, also denoted as “45, X” [31] or “45, XO”. The loss of chromosome X in the oocyte results from chromosome nondisjunction during meiosis. Phenotypically, Turner syndrome encompasses a spectrum of disorders. Girls may present with a webbed neck, wide-spaced nipples, flat chest, short stature, low hairline, cardiac and/or renal congenital anomalies, and gonadal dysgenesis causing primary or secondary amenorrhea. All women with Turner syndrome are prone to spontaneous ovulation. Natural pregnancy can occur in 2–7% and is associated with miscarriages, stillbirths, malformations, and chromosomal aberrations. Pregnancy in girls and young women with Turner syndrome is considered high-risk, hence discussion regarding contraception and family planning is recommended [32]. Turner syndrome is rarely inherited from mother to daughter and is more commonly a sporadic de novo genetic disorder. An early study evaluating the ovaries of eight 45, XO fetuses histologically, ranging in age from five weeks to four months [33], found no significant differences between XO and XX gonads up to the third intrauterine month. In the older fetuses, however, instead of the normal formation of primary follicles, there was a relative increase in the connective tissue of the gonad, suggesting that the defect of the XO ovary is not at the level of the germ cell but in surrounding follicular cells. As a result, germ cells entering the first meiotic prophase may not be able to organize PrFs, with degeneration occurring as a consequence. The histological findings of germ cells in the gonads of XO fetuses are starkly different from XO adult ovaries, which are usually devoid of germ cells. This study suggested that the etiology of POI in Turner Syndrome is from accelerated atresia of germ cells rather than a defect of germ cell formation or migration in utero.

Approximately 50% of all women with Turner Syndrome are thought to have a mosaic genotype. This mosaicism generally includes a 45, X cell line with another cell line of 46, XX, 47, XXX, or a number of other X-chromosome deletions [34]. These patients will exhibit a milder phenotype and might be diagnosed due to infertility [24]. The diagnosis of Turner Syndrome or mosaicism is done via karyotyping. Turner syndrome can be diagnosed during prenatal screening or if suspected in childhood, adolescence, or adulthood via a peripheral blood karyotype. If the karyotype is normal, but the level of suspicion is still high, the recommendation is to repeat the karyotype using a different tissue. This can help elucidate the patients with a mosaic genetic makeup. The percentage of mosaicism may be different in the ovary compared to the peripheral blood; hence, the phenotype does not always align with the karyotype. Nevertheless, it is not recommended to pursue an ovarian biopsy. In cases where a Y chromosome is present, there is an elevated risk of developing gonadal neoplasia (45%) [35]. Strikingly, Gravholt et al. [36] examined 114 females with Turner syndrome for the presence of Y chromosome material. Before this study, Y chromosome material was found in seven patients, but when repeated with PCR, it was found in 14 patients (about 12%). Overall, ten patients underwent ovariectomy, seven before entering the study due to verified Y chromosome material, and three due to the findings of this study. The histopathological evaluations showed that one of the ten ovariectomized patients had a gonadoblastoma. These results emphasize the importance of accurate karyotype.

*A. Primary Prevention*

Currently, there is no known prevention or treatment for Turner syndrome. Gonadectomy is recommended for all women with detectable Y chromosomal material [35].

*B. Secondary Prevention*

During prenatal care, ultrasound screening can detect specific features. Additionally, non-invasive prenatal testing (NIPT) might raise suspicion [37]. Conformation with prenatal diagnostic testing: CVS/amniocentesis and subsequent karyotype of fetal cells are available.

*C. Tertiary Prevention*

If diagnosed later in life, consulting with an REI to discuss the possibility of oocyte cryopreservation is valuable. Oocyte cryopreservation is possible only when ovarian follicles are present in mosaic Turner syndrome patients [38]. At present, there are no reports on the long-term outcomes from these cryopreserved oocytes. In the past, pregnancy for all women with Turner syndrome was a contraindication. It is now believed that there may be a subsect of women who can safely carry a pregnancy with multidisciplinary support. In the women for whom pregnancy is deemed safe, autologous or donor oocytes with embryo transfer are optional. If pregnancy is contraindicated, in vitro fertilization (IVF) with the transfer of an embryo into a gestational carrier is a possibility [39]. Another modality of fertility preservation that can be considered is ovarian tissue cryopreservation (OTC) [40]. OTC is pertinent when the patient is too young for a cycle of COH, or when medical treatment for an underlying disease cannot be delayed by a COH cycle, which takes about ten days to allow egg retrieval [41]. OTC, however, is an invasive technique and is limited to patients with good ovarian reserve.

#### 3.1.2. 47, XXX

Forty-seven, XXX syndrome (trisomy X) is another common cause of POI. It occurs in 1 in 1000 female births but estimated that only approximately 10% of cases are diagnosed. Most infants have a normal phenotype [42], and only a few cases with 47, XXX and congenital malformations were reported. Its most common physical features are high stature, epicanthal folds, hypotonia, and clinodactyly. One-third of affected female infants show some mental or behavioral problems. Seizures, motor and speech delays, cognitive deficits, learning disabilities, attention deficits, mood disorders, renal and genitourinary abnormalities, delayed menarche, and POI are also associated with this condition.

Forty-seven, XXX syndrome occurs mainly because of nondisjunction during meiosis, and about 20% occurs due to postzygotic nondisjunction. The phenotype in 47, XXX is hypothesized to result from the overexpression of genes that escape X-inactivation and the risk increases with advanced maternal age [43].

*B. Secondary Prevention*

During prenatal care, results from NIPT might raise suspicion [37] and is confirmed with prenatal diagnostic testing, CVS/amniocentesis, and subsequent karyotype of fetal cells.

#### 3.1.3. Other X Chromosome Anomalies

Other defects in the X-Chromosome can lead to Turner syndrome with or without mosaicism. One such defect is isochromosome Xq (46, X, i(X)q), where one X chromosome has two copies of the long arm of the X chromosome and is also monosomic for the short arm of the X chromosome. Ring chromosome X (rX) involves a ring forming when a portion of both; the long and short arms of X are missing. Any deletion of the long arm of X [del(X)q] or short arm of X [del(X)p] can also result in POI [44]. These anomalies all have varying phenotypic penetrance.

#### 3.1.4. Fragile X Syndrome

Fragile X syndrome (FXS) is an X-linked disorder and the most common inherited cause of intellectual disability in males and is a leading single-gene defect associated with an autism spectrum disorder. FXS is caused by a Cytosine-Guanine-Guanine (CGG) trinucleotide repeat expansion. The expansion is within the 5′ untranslated region of the fragile X messenger ribonucleoprotein 1 (*FMR1*) gene leading to a loss-of-function mutation. *FMR1* is located on the long arm of the X chromosome (Xq27.3) [45]. The number of CGG repeat expansions results in a partial or complete loss of gene expression of the encoded protein (FMRP). The expansion length correlates directly, but not linearly, to the severity of the phenotype. The American College of Medical Genetics and Genomic (ACMG) and American College of Obstetrics and Gynecology classified the CGG expansions into four categories: normal (5–44 repeats), intermediate or gray zone (45–54 repeats), premutation (PM) (55–200 repeats), and full mutation (FM) (>200 repeats) [46]. Patients with a FM have complete silencing of the *FMR1* and classic FXS phenotype [47]. In PM carriers, a portion of *FMR1* is transcriptionally active, and phenotypic expression has varying penetrance. Importantly, each subsequent generation has a risk of expansion of the CGG repeats. For example, intermediate carriers are at risk of having children with a PM, while PM carriers are at risk of passing on a FM to the subsequent generation. Fragile X PM carriers have a 20% lifetime risk of developing POI [48]. The exact pathophysiology is unknown but is thought to be secondary to an RNA-toxic effect [49]. Women with a FM or intermediate-sized CGG repeats are not at an increased risk of developing POI [50].

PM prevalence in the general population is 1:150–300 in females and 1:400–850 in males [51]. Significantly, Carrying the *FMR1* PM is more frequent among certain ethnic groups, highest in Colombia and Israel (1:100) and lowest in Japan (1:1674) [51,52]. As a result, in some countries (e.g., Israel), there is a recommendation for broad screening [53]. In other cases, without a known prior family history of FXS, the diagnosis is made based on clinical suspicion. When women present with POI, testing for FXS is recommended universally as a part of the workup. The molecular test evaluates the number of CGG repeats in the *FMR1* gene. Nolin et al. stratify the risk for expansion to a FM in the next generation, where beyond 100 CGG repeats the risk approaches 100%, and at 90 CGG repeats, is approximately 87% [54]. In PM carriers with an expansion of up to 90 CGG repeats, further analysis of the sequence to determine the number of AGG interruptions (AGG interruption testing), is recommended. This sequence analysis can help predict the likelihood of expansion to a FM in the offspring. The presence of AGG interruptions affects how likely the allele is unstable, how often it will expand to a FM, and the magnitude of expansion during transmission [55].

Interestingly, the degree of impact on the ovarian reserve varies according to the length of the CGG repeat sequence. Under 45 repeats, it is debated whether there is an impairment in the ovarian reserve [56,57]. Nevertheless, the AACMG’s official statement is that normal alleles have a range of 5–44 CGG repeats [58]. In the PM range as a group, the ovarian reserve is lower compared to non-carriers [59]. Women with midsize CGG repeats (about 80–100) are more prone to develop POI (FXPOI) [49]. When seeking ART, fewer eggs were retrieved from PM carriers compared to age-matched controls carrying less than 55 CGG repeats [60]. The response to ovarian COH is correlated with the number of CGG repeats [61,62]. More studies are needed in this population to evaluate long-term data on children born from IVF with preimplantation genetic testing (PGT)/ preimplantation genetic testing for monogenetic/single-gene diseases (PGT-M) for FXPM patients.

*A. Primary Prevention*

There is no known treatment for FXS at this point. Patients found to be with an intermediate, PM, or FM range should be offered further genetic testing [63]. A patient carrying a PM should be advised about the risks of allele expansion into a FM in the offspring, FXPOI, and fragile-X-associated tremor/ataxia syndrome (FXTAS). Some patients seeking care due to infertility are diagnosed as PM carriers during the evaluation, while others are diagnosed after giving birth to a child with FXS. When a woman is diagnosed as a PM carrier, she should be advised that other family members might also be affected. Female family members could be carriers, at risk of developing FXPOI and expansion to a FM in the offspring. Additionally, PM carriers are at risk of FXTAS, a neurological manifestation (usually with a late onset) with a phenotype that includes progressive cerebellar gait ataxia and intention tremor. Penetrance is increased with age, exceeding 50% for men aged 70–90 years, with women affected less severely and with lower penetrance. Women wishing to prevent passing a CGG expansion to their children can undergo IVF-PGT-M. Currently, there are two options for testing embryos. The first is by linkage analysis, which allows identification of inherited PM but yields no information about its length, expansion, or contraction. The second option is PGT-M for Fragile-X, which can determine the repeat number in the embryo. Many patients not only want to have an unaffected pregnancy, but also want to avoid passing on the PM, as it is not a benign state (risk for FXPOI and FXTAS); for this purpose, the linkage analysis is sufficient. As many FXPM carriers obtain fewer tested embryos, they may consider transferring “affected embryos” as a last resort, knowing that the risk for expansion is low. Utilizing PGT-M reveals the exact repeat number in each tested embryo and enables choosing embryos in the PM range for transfer.

*B. Secondary Prevention*

In countries where there is active screening or a patient is diagnosed due to reasons other than POI (a family member was diagnosed with a PM/FM), there are available strategies in the form of tertiary prevention.

*C. Tertiary Prevention*

Include oocyte cryopreservation in early adulthood or teen years in patients that are anticipated to become FXPOI. In some countries (e.g., Israel and Netherlands), PM carriers can cryopreserve their oocytes as a part of their healthcare policy regulations [64].

### 3.2. Single Gene Defects Causing POI

FMR1 PM is one of the most common single-gene defects leading to POI in 46, XX, women. Other single-gene defects associated with POI are listed below and grouped by the involved processes. These are mutations on autosomes or sex chromosomes and mitochondrial genes. Multiple gene mutations have been reported, but others are likely to exist.

#### 3.2.1. Transcription Factors

- Nuclear receptor subfamily 5 group A member 1 (*NR5A1*), a transcriptional activator involved in sex determination [65].
- Factor in Germline Alpha (*FIGLA*) regulates the expression of the zona pellucida and other oocyte-specific genes. Mutations in this gene are responsible for about 4% of POI cases [66].
- Newborn Ovary Homeobox (*NOBOX*), a mutation in that gene, is responsible for about 6% of POI patients [67].
- Forkhead Box L2 (*FOXL2*) is a specific transcription factor that binds a DNA domain; a dominant mutation will cause POI [68]. Mutation in *FOXL2* causes haploinsufficiency and is also the base for Blepharophimosis, Ptosis, and Epicanthus Syndrome (BPES), Which has a POI phenotype [69].
- Diaphanous Homolog 2 (*DIAPH2*) has a role in the development and normal function of the ovaries [70].

#### 3.2.2. Growth Factors

- *BMP-15* [71].
- Growth differentiation Factor 9 (*GDF-9*) [72].
- Inhibin Alpha Subunit (*INHA*) [73].

#### 3.2.3. Steroidogenesis

- *FSH* [74].
- FSH Receptor (*FSHR*) [75].
- LH Receptor (*LHR*) [76].

#### 3.2.4. Galactosemia

A rare autosomal recessive disorder due to deficiency in any one of the three enzymes. One of them, galactose-1-phosphate uridyltransferase (GALT), is related to POI. More than 80% of girls and women with classic galactosemia have POI, regardless of diagnosis at birth, and strict galactose restriction in their diet [77]. The exact mechanism is not clear, however, studies examining the influence of high-galactose diet on pregnant rats found a striking reduction in oocyte number in offspring [78] as well as impairment of germ cell migration [79]. In humans, toddlers that are galactosemic show abnormally low AMH levels when compared to age-matched controls [80].

*B. Secondary Prevention*

For galactosemia patients, genetic counseling regarding the risk of infertility; and a pediatric endocrinologist follow-up are recommended. In prepubertal patients, OTC [81] may be considered. If no ovarian activity is detected or COH fails, oocyte or embryo donation at the time the patient would like to build her family is optional [82]. 

#### 3.2.5. Mitochondrial DNA Polymerase Gamma (POLG)

DNA polymerase responsible for mitochondrial DNA replication and repair, thus essential for mitochondrial function. Gene mutations were reported to be correlated to early menopause by Luoma et al. [83] and later association with POI.

### 3.3. Idiopathic

While idiopathic POI, by definition, means there is no known cause, it is thought to have an underlying genetic component. This group is presumably the largest; idiopathic cases are reported as high as 50–90% of POI patients, with a 44–65% link in mother and daughter pairs and 10–30% of cases having an affected first-degree relative. Additionally, women with an affected mother are six times more likely to develop POI [84]. This points to a possible underdiagnosis of genetic causes of POI in the idiopathic population. Genome-wide association studies (GWAS) have implicated several loci in family lines [85]. If a familial association is suspected, work-up with karyotype or other better-known gene mutations is negative, GWAS remains an option to investigate the presence of a lesser-known gene mutation among families [86].

*B. Secondary Prevention*

ESHRE recommends that all women with non-iatrogenic POI should perform chromosomal analysis [3]. However, further studies are needed in these lesser-known idiopathic cases with a genetic influence.

There is no broad recommendation for broad screening for genetic disease. Nonetheless, where a known genetic background exists in discrete families, it is advised to test. As part of prenatal care, there are screening panels for genetic diseases, some of which can lead to infertility or subfertility. Early diagnosis could help the patient decide toward fertility preservation or change their family-building plans. Pan-ethnic carrier screening before IVF treatment is recommended in some medical centers. Occasionally, when utilizing these tests, an unpredictable diagnosis is made. Identification of a genetic mutation could change the patient’s plan for conceiving or induce other family members to be tested, as some genetic mutations are familial. The pan-ethnic carrier screening test tests roughly 400 genes; mutations in these genes can cause morbidity or mortality (e.g., cystic fibrosis, sickle cell disease, thalassemia, and spinal muscular atrophy). Most of the tested conditions are autosomal recessive, so it may be necessary for the partner to be tested as follow up. Accordingly, a recommendation regarding the use of in vitro fertilization-pre-implantation genetic diagnosis (IVF-PGD) is imperative. Briefly, IVF patients will undergo COH, retrieval of mature eggs, fertilization of the eggs by sperm, and development of embryos. The embryos will be biopsied, and the biopsied cells will be tested. This enables the selection of unaffected embryos for transfer.

As part of prenatal care, genetic counseling for couples is advised. In that consultation, the medical history of both partners’ families will be looked at, and if indicated, genetic testing will be recommended. In cases where an unknown condition is detected, for example, carrying a premutation of fragile X, a recommendation for all family members to be tested is advised since this condition can affect fertility in female family members without other phenotypic traits. In this event, affected family members may consider follow-up and fertility preservation if warranted.

## 4. Infections

While infectious etiologies for POI are listed in multiple reviews (shigellosis, chickenpox, mumps oophoritis [87,88], tuberculosis (TB)- pelvic tuberculosis, malaria, and cytomegalovirus infection), their true incidence is undetermined. The global burden of TB is high; its incidence is estimated at 10 million per year [89]. More than 1.7 billion people (approximately 22 percent of the world population) are estimated to be infected with M. tuberculosis, and about 95 percent of TB cases occur in resource-limited countries. The dramatic differences in prevalence between countries and continents may be related to the fact that there is less available scientific evidence about POI. Most publications focus on pelvic inflammation as a cause of infertility due to TB and not directly a cause of POI. There is evidence of reduced ovarian reserve, with lower AMH levels and AFC counts, but not specifically POI, and the underlying mechanisms are unclear [90]. Hypothetically, medical treatment for chronic infections like TB, malaria, etc., could be correlated with POI. More research is needed to clarify whether there is an increased risk for POI in those infectious diseases and what the underlying mechanisms are.

*A. Primary Prevention*

There are effective vaccines available for chickenpox and mumps.

*B. Secondary Prevention*

There is no indication to screen for infectious etiologies in a woman with POI without noted risk factors [3].

### 4.1. Human Immunodeficiency Virus (HIV) Infection

People with HIV live longer with a better quality of life due to dramatic advances in antiretroviral therapy. This also allows women with HIV to build families. Most women infected with HIV contract it during their reproductive years; thus, its effect on the ovarian reserve is highly relevant. The level of evidence for an influence on ovarian reserve in women living with HIV (WLWH) is limited. Cejtin et al. reported that HIV seropositive women were three times more likely than seronegative to enter menopause earlier [91] and the infection itself may be associated with amenorrhea [92]. Other studies have correlated HIV infection in untreated patients with subfertility, however, this could be explained by the co-occurrence of other sexually transmitted infections (STI), some of which can lead to pelvic inflammatory disease (PID), malformation/scarring of the fallopian tubes, and tubal factor infertility that does not affect the ovarian reserve [93]. Indeed, women infected with HIV are prone to more severe clinical manifestations of PID [94]. Finally, some WLWH use or have used recreational drugs that could reduce the ovarian reserve [95].

A direct link between HIV infection and diminished ovarian reserve remains to be established, however, using serum AMH as a proxy, different groups found both higher [96] and lower [97,98] AMH levels in WLWH. Santulli et al. showed that viral load was associated with decreased serum AMH levels, and an increase in CD4⁺ cell count was directly associated with serum AMH levels [98]. Such inconsistencies make it hard to define whether there is a correlation between HIV infection and ovarian reserve. Regardless, there is support in the literature that HIV infection is associated with an inflammatory phenotype, chronic immune activation, immune senescence [99,100], and oxidative stress [101]. As people with HIV live longer, mitigation of the toxic influence on the ovary is critical.

*A. Primary Prevention*

The primary level of prevention is keeping people from contracting disease. HIV is transmitted through body fluids: Blood, pre-seminal and seminal fluid, rectal fluids, vaginal and cervical fluids, breast milk, and during pregnancy and childbirth. Additionally, contact between mucosal tissue or damaged tissue and contaminated fluid is a risk factor. Regular use of barrier contraceptives like condoms or taking medications prevents or treats HIV; pre-exposure prophylaxis (PrEP) reduces the risk of HIV infection via sex or injection drug use.

*B. Secondary Prevention*

Prevention of transmission by people who live with HIV to people who are negative for HIV. Education of people living with HIV on reducing the risk of HIV transmission. Supporting patients’ adherence to their HIV medication to maintain viral suppression and prescribing PrEP for HIV-negative sexual or drug-injecting partners is also advised.

*C. Tertiary Prevention*

Providing medical and psychiatric interventions as soon as possible to help women manage and live with the disease [102].

### 4.2. Severe Acute Respiratory Syndrome Coronavirus 2 (SAR-CoV-2) Infection

In December 2019, coronavirus disease 2019 (COVID-19) was first detected in Wuhan, China. COVID-19 is caused by severe acute respiratory syndrome coronavirus 2 (SAR-CoV-2), a novel coronavirus, and was declared a global pandemic in March 2020, spreading rapidly, and affecting millions worldwide. While morbidity and mortality resulting from COVID-19 were grave and significant health risks remain, concerns related to female fertility arose after women noted abnormal patterns in their menstrual cycles [103].

The primary mechanism by which the SAR-CoV-2 enters host cells is the attachment of its spike protein to the angiotensin-converting enzyme 2 (ACE2) receptor [104]. After attaching, the viral genome and nucleocapsid are released into the host cell cytoplasm [105], followed by cleavage of the S protein by transmembrane serin protease-2 (TMPRSS2); and fusion of the cellular membranes of the host and virus [104]. The ACE 2 receptor is present in the human ovary and can be a potential docking point for the virus, however, expression of TMPRSS2 measured in 18 human cumulus cell samples showed transcript levels to be low or absent. These analyses suggest that SARS-CoV-2 infection is unlikely to have long-term effects on female reproductive function [106] and another study found that cumulus and granulosa cells show low susceptibility to SARS-CoV-2 infection due to a lack of the required combination of receptors and proteases (ACE2/TMPRSS2) in high abundance [107].

Evaluation of ovarian reserve in the context of COVID-19 using the AMH measurement as a proxy has revealed contradictory evidence. Li et al. found no difference in AMH levels when comparing women who contracted COVID-19 to controls [108], and Bentov et al. showed no impact of SARS-CoV-2 infection and/or COVID-19 vaccination on ovarian function [109]. On the other hand, Ding et al., found lower serum AMH levels in women hospitalized due to COVID-19 compared to matched controls [110]. Overall, it seems that the infection and vaccination do not affect the ovarian reserve, nevertheless, the full extent of their influence on ovarian reserve and the ramifications for fertility may not yet be apparent. Moreover, the rapid mutability of the virus may introduce variants with different receptor affinities that result in a greater impact in the ovary.

## 5. Autoimmune Disease

Roughly 30% of POI cases are autoimmune by nature [111,112], however, investigating autoimmune disease is challenging due to the scarcity of ovarian tissue for research. While animal models exist [112], the pathogenesis of autoimmune POI remains poorly defined. There is a correlation between histologically diagnosed oophoritis and circulating adrenal or ovarian steroid-cell autoantibodies (SCA) that target steroidogenic enzymes [112]. There is no documentation of infiltration of ovaries by immune cells without SCA presence. Thus, there is no indication for an ovarian biopsy to diagnose autoimmune POI and testing for SCA in the serum is sufficient. Circulating SCA can be detected even years before clinical diagnosis of POI. In the literature, there are also case reports on antibodies against LHR [113], FSHR [114], and zona pellucida [115]. No validation and diagnostic accuracy of the antibody assays has been published; hence, their specificity and pathogenic role remain uncertain. It is possible that a serological marker may not be detected at the time of diagnosis regardless of its autoimmune etiology, either due to the waning of the immune response or low exposure [116]. Clinical data suggest that a substantial proportion of autoimmune POI is an isolated disease, while the rest co-exist with other autoimmune diseases. They include endocrine diseases: Thyroid, adrenal (Addison’s disease), hypoparathyroidism, diabetes mellitus type 1, and hypophysitis; and non-endocrine diseases: chronic candidiasis, immune thrombocytopenic purpura, vitiligo, alopecia areata, autoimmune hemolytic anemia, pernicious anemia, systemic lupus erythematosus, rheumatoid arthritis, Crohn’s disease, Sjögren’s syndrome, multiple sclerosis, myasthenia gravis, primary biliary cirrhosis, celiac disease, inflammatory bowel diseases, glomerulonephritis, and chronic active hepatitis [112,117,118,119,120,121].

A less frequent autoimmune condition that also involves ovarian failure is polyglandular autoimmune syndrome (PAS-2). There are two histological phenotypes for this condition: the first (about 60% of patients) presents as an isolated oophoritis with fibrosis and absence of follicles; in the second group, ovarian follicles are present. Addison’s disease is a frequent autoimmune condition estimated in 2–10% of POI patients [119]. It is speculated that SCA in these patients target shared autoantigens between adrenal and ovarian steroid-producing cells. Histological findings of lymphoplasmacellular infiltration around steroidogenic cells provides evidence for immune system involvement. POI of adrenal autoimmune origin is the most frequent type observed in 60% to 80% of patients with autoimmune POI [122]. These women should be counseled and screened for POI. The second most common autoimmune POI, without adrenal involvement, is thyroid autoimmunity (14–27%) [112,123]. Yi-Ting Hsieh et al. published a longitudinal population-based retrospective cohort study that concluded that thyroid autoimmunity significantly increases the risk of POI in women [124]. This study warrants the need for ovarian reserve evaluation among these women.

*B. Secondary Prevention*

While there is no specific treatment option, identifying women with autoimmune POI is clinically relevant. It is pertinent for diagnosing patients with subclinical or latent autoimmune Addison’s disease. Special consideration is needed in cases where the second line of treatment includes chemotherapeutic drugs that are gonadotoxic; in those patients, consultation with an REI is advised.

*C. Tertiary Prevention*

It is important to evaluate the ovarian reserve in women diagnosed with autoimmune disease due to the coexistence of related conditions. In the initial phase of autoimmune POI, ovarian reserve is still normal, allowing for the cryopreservation of gametes.

While not clinically available at present, in vitro folliculogenesis or oocyte maturation could be applied to cryopreserved or fresh ovarian tissue. Culturing ovarian tissue or follicles, isolated from the deleterious effects of the immune system, could yield mature oocytes for cryopreservation and/or fertilization.

## 6. Medical Treatment (Chemotherapy, Radiation, and Surgeries/Procedures)

Iatrogenic causes of POI are more common as access to treatment and surgery for benign and malignant diseases increases. Fortunately, with improved treatments, childhood cancer survival rates are up to 80%. These girls and young women completing their cancer treatments: chemotherapeutic agents and/or radiation are at an increased risk of developing POI later in life [125,126]. Other risk factors for POI are the surgical management of reproductive cancers, prophylactic surgeries, resection of ovarian cysts, and endometriosis [127,128,129].

### 6.1. Chemotherapy

Women diagnosed with cancer until the age of 39 years are 38% less likely to achieve a subsequent pregnancy [130,131], presumably due to the detrimental effects of chemotherapy on the ovarian reserve. Chemotherapeutic agents, especially alkylating agents, are gonadotoxic for girls and pre-menopausal women and significantly reduce ovarian reserve through myriad biological pathways which are not fully understood [132,133]. The degree of gonad toxicity in different treatments depends on the type of compound (alkylating vs. non-alkylating), frequency, duration, and age when treated [126]. In addition, chemotherapy is used for treating non-malignant diseases, such as auto-immune disease, and as part of a conditioning regimen for stem cell transplants [134,135]. Chemotherapy is administered in different combinations, combined with other drugs, radiation, or surgery. For this reason, it is challenging to isolate the exact impact that each agent has on ovarian function and fertility [136].

Alkylating agents (e.g., cyclophosphamide and busulfan) covalently bind alkyl groups to DNA, induce DNA crosslinking, and prevent effective DNA replication. Initial observations after administration of cyclophosphamide were decreased ovarian reserve and amenorrhea [137,138,139]. In rodents, exposure to cyclophosphamide reduces the PrF count in a dose-dependent manner [140]. Patients treated with alkylating agents compared to other gonadotoxic chemotherapeutics had a significant reduction in PrF numbers [141]. Another class of drugs, platinum-based compounds (cisplatin and carboplatin), also promote DNA crosslinking, interfering with DNA repair mechanisms blocking cell division, and ultimately triggering apoptotic cell death, particularly in rapidly dividing cells. These drugs have a milder effect on the ovarian reserve [142,143]. In rodent models, cisplatin induces DNA damage in quiescent PrFs [144,145] and directly activates PrFs leading to loss of the ovarian reserve [146]. Human studies have shown contradicting results regarding the effects of cisplatin or other platinum-based therapies on PrF numbers, primarily because these drugs are administered as part of a combined regimen [147,148]. Another class of chemotherapy, anthracyclines (doxorubicin), damage the ovarian reserve, but to a lesser extent. Anthracyclines induce the nuclear enzyme topoisomerase II and its ability to intercalate DNA. It leads to the cellular accumulation of DNA fragments during replication and cell death. Even though animal models and in vitro studies have shown that doxorubicin has a detrimental effect on the ovarian reserve [149], it is not considered a high-risk drug for POI [126]. More subtle effects of other chemotherapeutic drugs, such as taxanes, are possible but remain uncertain.

The mechanisms by which chemotherapies lead to a reduction in the ovarian reserve are still not fully understood, and more than one pathway may be involved in this response. Studies have found that the loss of PrF count is secondary to the loss of growing follicles [150]. Growing follicles secrete AMH from granulosa cells; when their number decreases, less AMH is secreted, resulting in less inhibition of PrF activation, and PrFs that are activated undergo atresia [126,146,150,151]. This effect seems to be modulated, at least partly, via the PI3K/PTEN/Akt pathway [152], which may also be activated directly by chemotherapy [150,153]. Several studies have found direct DNA damage in PrFs secondary to chemotherapy [154,155,156], leading to the activation of pro-apoptotic pathways and subsequent PrF loss [157]. In addition, these therapies can detrimentally affect ovarian stroma, particularly endothelial cells [157,158], resulting in a reduction in blood flow to the ovary, fibrosis, impaired ovarian function, and potentially further damage to the reserve [159,160].

A rapidly expanding category of cancer treatment is immunotherapy, which has become the standard of care for many malignancies, however, the potential effects of these treatments on ovarian reserve and infertility were not thoroughly investigated before their approval. Winship et al. has since evaluated whether immune checkpoint inhibitors action of blocking programmed cell death protein ligand 1 and cytotoxic T lymphocyte-associated antigen four has an influence on mice ovaries. Strikingly, immune cell infiltration and tumor necrosis factor-α expression were increased within the ovary, resulting in diminished ovarian reserve and impaired maturation and ovulation of oocytes [161].

*A. Primary Prevention*

When applicable, a treatment combination that is the least gonadotoxic is preferable. Understanding the underlying mechanisms involved in PrF loss due to chemotherapy is a prerequisite to developing therapeutics that can ameliorate their gonadotoxic effects. The American Society for Reproductive medicine (ASRM) recommended, in a committee’s opinion, a multidisciplinary collaboration between oncologists and reproductive specialists [41]. The committee emphasized that despite increasing awareness regarding fertility preservation, its importance, and published recommendations, counseling and services remain underutilized. It is imperative to improve collaboration between oncologists and reproductive specialists and expand the availability of and access to fertility-preservation services for patients facing fertility-threatening therapies [162].

Current fertility preservation techniques, cryopreserving oocytes, embryos, and OTC when the ovarian tissue is free of residual disease [163,164], are available and used widely for that purpose. In a large-scale comparison of outcomes between patients with age-related elective oocyte cryopreservation to egg freezing before chemotherapy (most patients had breast cancer or lymphoma-both Hodgkin’s and non-Hodgkin’s), success rates were lower in cancer patients. Nonetheless, there was no statistically significant association between malignant disease and reproductive outcome after correction for age and COH regimen [165]. When comparing different cancer types, the ovarian response was not affected by the type of cancer, but there was a reduction in the oocyte yield after ovarian surgery [166]. Embryo cryopreservation is a well-established method for preserving fertility, but it has limitations. Following fertilization of an oocyte, it carries the genetic material of both partners, limiting options for the patient. Cryopreservation of mature oocytes addresses this, and vitrification is a widely used [167] and straightforward procedure for providing the patient reproductive autonomy [168]. Indeed, a systematic review and meta-analysis by Rienzi et al. showed that vitrification is superior to slow freezing for cryopreservation of human oocytes and embryos in clinical ART [167].

While application of fertility preservation is expanding, it is not always available due to lack of access, advanced facilities, cost, or urgency in commencing chemotherapy. For these reasons, many groups have focused on developing therapeutic medicines to ameliorate chemotherapy on the ovarian reserve [169]. One of the strategies, the use of gonadotropin-releasing hormone (GnRH) analogs for ovarian protection during chemotherapy, is controversial. The speculated mechanism is that GnRH induces the release of pituitary FSH and LH. Desensitizing the pituitary to its effect during chemotherapy, blocking FSH increase, and the accelerated recruitment of follicles [169]. Results regarding the fertoprotective effects of GnRH analogs in a clinical setting are varied, with moderate benefits found in some cohorts of breast cancer patients [170,171], but with other studies not finding a benefit for fertility preservation [171,172]. The available studies offered limited follow-up, and endpoints were not a direct assessment of fertility, but only proxies, for example, using menstruation as an endpoint instead of live birth rate. There are clinical scenarios in which GnRH administration is useful: in cases where there is significant vaginal bleeding due to chemotherapy-induced thrombocytopenia, the administration will help control the bleeding.

Tamoxifen protects ovarian reserve from chemotherapy-induced damage in animal studies [173], however, no significant protective effect on the ovarian reserve was noted in patients treated with cyclophosphamide [174]. AS101, an immunomodulator of the PI3K/PTEN/Akt pathway, has also been applied in mice and resulted in a reduction of chemotherapy induced Akt phosphorylation with reduced PrF activation and decreased granulosa cell death [150]. Others have shown that modulation of mTOR signaling via inhibitors everolimus, INK128 [175], and rapamycin [175] had a protective effect on the ovarian reserve when given to chemotherapy-treated rodents. Another approach has focused on protecting the vasculature of the ovary using compounds such as S1P and ceramide-1 phosphate that promote vascular angiogenesis. Animal [176] and human xenograft [155] studies have shown that these factors reduce follicle death and ameliorate the vascular effects of chemotherapy. Similarly, granulocyte-colony stimulating factor (GCSF) increases micro-vessel density, decreases chemotherapy-induced ovarian follicle loss, and extends time to POI in female mice [177]. Another strategy for diminishing the effects of chemotherapy on the ovary is targeting of enzymes involved in drug transport and uptake, such as the multidrug resistance gene 1. It has been shown that its overexpression in transduced granulosa cells in animal models has protective effects [178]. AMH, or its analogs [179], have also emerged as a promising therapeutic to protect the ovarian reserve during chemotherapy [180]. AMH is secreted by granulosa cells of growing follicles, and it is thought to inhibit PrF recruitment and thereby contribute to maintenance of ovarian reserve [180,181]. Animal studies have shown that the administration of AMH in conjunction with chemotherapy protects ovarian reserve by preventing PrF activation [181,182]. While all these aforementioned pharmacologic and biologic modes of fertoprotection have promise, a major obstacle to their clinical translation is the need for repeated and/or continuous administration, in most cases directly to the ovary. While animal studies have explored ovarian/intrabursal delivery or continuous administration via osmotic pumps or genetically engineered cells, in humans these techniques are invasive, less practical, and have potential to contribute to undesired off-target effects. Hence, the development of targeted delivery mechanisms is a critical prerequisite to clinical applications.

*C. Tertiary Prevention*

Patients can cryopreserve oocytes, embryos, or ovarian tissue after the administration of chemotherapy, particularly in cases where treatments are only mildly damaging. Shapira et al. [183] demonstrated cryopreservation following chemotherapy in a high-risk case for residual diseases like leukemia; ovarian tissue from this patient was cryopreserved after complete remission, but before bone marrow transplantation. For this patient, tissue recovery during complete remission, coupled with intense tissue evaluation before transplantation, allowed for safe, successful transplantation in a survivor of acute myeloid leukemia.

### 6.2. Radiation

Ionizing radiation is used as a first-line treatment for many types of cancer and as a conditioning regimen for bone marrow transplantation in addition to alkylating agents. Radiation causes direct DNA damage, stalling of cellular division, and cellular apoptosis. Given its mechanism of action, irradiation is extremely gonadotoxic, leading to the death of exposed oocytes within a few hours [184]. The effects of radiation on the ovaries depends on the location, dosage, and patient age [185]. There is a direct correlation between patient age and the degree of ovarian damage, presumably due to the limited ovarian reserve available during the treatment. The older the patient is, the greater her risk of developing POI, however in young girls, POI may also occur after treatment with high doses and/or prolonged exposure. Oocytes are highly radiosensitive, with an LD50 of < 2 Gy [184]. In childhood cancer patients, POI often occurs when treated with a dose > 20 Gy [186,187], however, oocyte radio sensitivity depends on its growth phase, with PrF being more radio-resistant compared to growing follicles. Independent of the influence on follicles, radiation also affects the vasculature in the stroma of the ovary, leading to atrophy and fibrosis [126].

*A. Primary Prevention*

When the irradiation field excludes the pelvic area, there is a low chance of POI secondary to radiation Tx [188]. For this reason, elective laparoscopic ovarian transposition away from the pelvis before irradiation (oophorpexy) is recommended in patients with pelvic cancer [189]. Currently, the established protective treatments for irradiation to the pelvis or total body irradiation are oocyte/embryo cryopreservation, OTC [190], and surgical transposition (oophorpexy) of the ovaries away from the field of radiation [191,192,193,194,195].

### 6.3. Surgical

Surgical causes of POI remain the most common within the iatrogenic category [196]. Surgeries within the pelvic region and the gonads that result in insults to the ovarian cortex or reduced blood flow, as well as the complete removal of one or two of the ovaries, reduce the ovarian reserve [197]. Uterine artery embolization may reduce blood supply to the ovaries creating a pro-inflammatory environment in the pelvic area and can lead to transient [198] or permanent [199] ovarian failure, especially in women 45 years old and above. Surgical management of cysts or ovarian endometriotic lesions were found to have a detrimental effect on the ovarian reserve [200,201,202] secondary to damaging the surrounding healthy ovarian cortex. Studies have found that endometriomas (ovarian cystic lesions that stem from the disease process of endometriosis) directly lead to PrF activation [203] causing lower PrF density in the surrounding cortex [204]. Surgical management of ovarian endometriomas has been controversial [205], given that their removal is technically challenging and may damage the surrounding tissue [206]. In addition, endometriomas have a 10% incidence of recurrence after a year of removal surgery [207]. Accordingly, endometriomas have increasingly become a reason for fertility preservation [163]. Ovarian wedge resections and ovarian drilling, a technique historically used to treat polycystic ovarian syndrome has been found to reduce the ovarian reserve [208] due to direct damage of the surrounding ovarian cortex and/or interruption of blood supply to the ovary. The loss of growing follicles results in a drop in AMH levels following surgery [209,210,211], thereby enable a permissive environment for increased follicular recruitment and depletion of the ovarian reserve. In support of this, animal studies have shown PrF activation via the mTOR pathway in response to surgical insult [212] and multiple analyses have suggested that unilateral oophorectomy leads to earlier onset of menopause [213,214,215].

*A. Primary Prevention*

Before a surgical procedure that may affect the ovary, patients should be counseled on the possible effects of the surgery on the ovarian reserve. When possible, the least damaging technique for the ovarian reserve should be used. If recommended and desired, gamete banking or OTC are available. Prophylactic surgical intervention that might affect the ovarian reserve should be carefully considered by physicians.

## 7. Environmental Exposure

It is challenging to study the effects of different environmental pollutants related to POI, considering the general population’s exposure to a combination of chemicals from food, personal hygiene and cosmetic products, the environment, etc., but these factors can contribute to DOR in adulthood.

Environmental pollutants are thought to affect the ovarian reserve through three main pathways: endocrine activity; commonly referred to as endocrine-disrupting chemicals (EDCs), oxidative stress, and epigenetic modifications [216].

Few studies have been able to correlate environmental exposure to pollutants and the onset of POI in humans. However, animal studies suggest that pesticides, cigarette smoking, and phthalates are associated with POI. One exposure with known links to infertility is cigarette smoking. According to the CDC, about 11% of women smoke and 21% of these are in their reproductive years, ages between 18 and 44 [217]. Tobacco smoke contains hundreds of potentially harmful substances: nicotine, carbon monoxide, and recognized carcinogens and mutagens [218]. A relationship between cigarette smoking and early menopause was noted more than 40 years ago [219]. Later, it has was shown that there is an effect on both time to conception and spontaneous abortion risk [220], as well as a decreased success rate in the context of ART [221]. In mice, it has been shown that cigarette smoke has an adverse effect on the ovarian reserve [222], but the mechanisms underlying this effect remain to be elucidated. In humans, the available data is limited and contradicting. El-Nemr et al., found in a retrospective study, that women who smoked had a higher serum FSH and required a higher mean dosage of gonadotrophins for COH than non-smokers [223]; and reduced AMH levels in active smokers in late-reproductive-age and perimenopausal years has been observed [224]. However, a recent prospective study found no significant difference in quantitative ovarian reserve markers between current smokers, ex-smokers, and never-smokers [225]. This was consistent with a previous study that concluded neither smoking status nor second-hand smoke exposure in utero, childhood, or adulthood was associated with AMH levels [226].

### 7.1. Endocrine Disrupting Chemicals

The Endocrine Society defines EDCs as an exogenous chemical, or mixture of chemicals, that interferes with any aspect of hormone action. The effects of EDCs on ovarian function can either be permanent or transient, depending on exposure time [227].

#### 7.1.1. Phthalates

Phthalates are materials included in manufacturing plastics and pesticides and are considered to be risk factors for POI. Multiple phthalates have been identified as EDCs and been shown to tamper with reproductive functions and endocrine signaling. Human exposure to phthalates can occur through oral ingestion, inhalation, or dermal exposure and products containing phthalates include medical, personal, and building products [228]. Multiple studies have demonstrated diminished follicle count after exposure to phthalates in mice at different ages, ranging from neonatal to adult life [229,230]. A study conducted on 215 infertile patients revealed a decreased AFC count in patients with higher urinary phthalate levels. Specifically, Di-2-Ethylhexyl phthalate (DEHP), a factor previously shown to accelerate PrF recruitment and arrest antral follicle growth, was increased [231].

#### 7.1.2. Pesticides

Some pesticides can be classified as EDCs and have a deleterious influence on both reproductive and ovarian function [216]. In murine studies, certain insecticides have been linked to a decrease in follicle count [232], herbicides can induce decreased ovarian weight [233], and exposure to EDC during development can alter the epigenetic programming and result in adult-onset disease [234]. In spite of this evidence, a study of 8038 American women living on farms did not find evidence that the use of specific pesticides led to earlier menopause. On the contrary, the use of hormonally active pesticides was associated with a later onset of menopause (median age increased by 3–5 months) [235]. In contrast to the results of this study, cross-sectional analyses have shown an association between organochlorides [236] and 1,1-Dichloro-2,2-bis 4-chlorophenyl ethene [237], two types of pesticides, with an earlier age of menopause.

Other organic pollutants have also been linked to reduced ovarian reserve: Exposure to polycyclic aromatic hydrocarbons (PAHs), a component of cigarette smoke, increases the risk of developing POI [238], and although no causal relationship has been proven between cigarette smoking and POI, it is associated with early menopause [216]. In a case-controlled study of 347 women, exposure to PAHs was not only associated with increased risk of POI, but also higher serum levels of FSH, and LH, and lower levels of AMH. This study suggested that exposure to PAH risks reproductive health and should be limited [239]; similar results were found in serum levels and were associated with the number of pack years [216].

### 7.2. Oxidative Stress

Oxidative stress relates to the overproduction of reactive oxygen species (ROS). Overproduction and elevated levels of ROS in ovarian cells lead to antral follicle apoptosis. In a cross-sectional study evaluating the levels of ROS in infertile women, inducible nitric oxide synthase (INOS), myeloperoxidase (MPO), and total oxidative status (TOS) were higher compared to healthy fertile women [240].

### 7.3. Epigenetic Modifications

Environmental pollutants can lead to changes in DNA methylation. Plusquin et al., found data to support that global hypomethylation is associated with air pollution [241], which can be inherited by future generations and may harm ovarian function [216].

*A. Primary Prevention*

Lifestyle modification is a robust mode of primary prevention of POI. Especially when addressing cigarette smoking, it is highly recommended to quit, not only from the standpoint of fertility and ovarian reserve, but for general health. In cases of exposure to hazardous chemicals at their workplace, women should be educated about the risk and adequately protected, particularly if they plan to conceive or are already pregnant.

*B. Secondary Prevention*

INOS, MPO, and TOS have been proposed as potential serum markers of POI [240]. Currently this is not recommended, but if tested and verified, these markers would provide a practical means of screening women to offer early intervention.

*C. Tertiary Prevention*

When POI is a by-product of hazardous exposure and the source is known, it should be discontinued. Additionally, fertility preservation should be considered in cases when there is still a substantial ovarian reserve.

## 8. Conclusions

This review elaborates on the etiologies and known mechanisms of premature ovarian insufficiency. The approach of the review was to focus on an actionable intervention available to prevent POI in a three-tier level of intervention; primary, secondary, and tertiary.

The primary prevention recommendations include lifestyle modifications that are beneficial for all; not smoking, avoiding exposure to toxins, vaccinations, etc. The recommendations for secondary prevention are more diverse and include fertility preservation techniques that underscore the importance of counseling and education regarding the impact of treatments and interventions available for preserving reproductive options. Unfortunately, there are currently no screening programs in place for the general population, however, we have highlighted situations in which screening is recommended (e.g., known familial genetic condition or chronic disease).

In general, combining clinical practice with increased awareness will enable diagnosis at a time when significant ovarian reserve remains, and fertility can be preserved. Increased education, along with a deeper understanding of the underlying mechanisms and development of treatments, will improve the outcomes for these patients (Table 1).

## Figures and Tables

**Table 1:** Summary.

Insult	Primary Prevention	Secondary Prevention	Tertiary Prevention
2. Developmental abnormalities	Limiting/avoiding chemical exposures during pregnancy. Emphasis on the avoidance of endocrine disruptors.	Genetic screening with genetic counseling when there is a family history of genetic abnormalities, developmental abnormalities, or POI. Considering prenatal diagnosis with CVS or amniocentesis.	Oocyte cryopreservation in early adulthood or teen years.
3.1.1. Turner Syndrome	Gonadectomy is recommended when Y chromosomal material is detected.	Might be diagnosed during prenatal care, detection of specific features by ultrasound screening or NIPT. Conformation with CVS/amniocentesis and subsequent karyotype of fetal cells.	Oocyte cryopreservation and OTC are possible only when ovarian follicles are present in mosaic Turner syndrome patients. If pregnancy is deemed safe, autologous or donor oocytes with embryo transfer are optional. If pregnancy is contraindicated, considering IVF with the transfer of an embryo into a gestational carrier.
3.1.2. 47, XXX		NIPT results might raise suspicion and is confirmed with CVS/amniocentesis, and subsequent karyotype of fetal cells.	
3.1.4. Fragile X Syndrome	When a woman is diagnosed as a PM carrier, advising her that other family members might also be affected. Women wishing to prevent passing a CGG expansion to their children can undergo IVF-PGT-M.	In cases a woman was detected as a carrier, there are available strategies for fertility preservation.	Oocyte cryopreservation in early adulthood or teen years in patients that are anticipated to become FXPOI.
3.2.4. Galactosemia		Genetic counseling regarding the risk of infertility. In prepubertal patients, OTC can be considered. If no ovarian activity is detected or COH fails, oocyte/embryo donation at the time the patient would like to build her family is optional.	
3.3. Idiopathic		Women with non-iatrogenic POI should perform chromosomal analysis. Where a known genetic background exists in discrete families, it is advised to test. As a part of prenatal care, using screening panels for genetic diseases. Early diagnosis could help the patient decide toward fertility preservation or change their family-building plans. Pan-ethnic carrier screening before IVF treatment is recommended in some medical centers. Identification of a genetic mutation could change the patient’s plan for conceiving or induce other family members to be tested. According to the results, a recommendation regarding the use of IVF-PGD is imperative.	
4. Infections	Chickenpox and mumps vaccines		
4.1. HIV infection	Regular use of barrier contraceptives like condoms or taking PrEP, to reduce the risk of HIV infection via sex or injection drug use.	Education of people living with HIV on reducing the risk of HIV transmission. Supporting patients’ adherence to their HIV medication to maintain viral suppression and prescribing PrEP for HIV-negative sexual or drug-injecting partners.	Providing medical and psychiatric interventions as soon as possible to help women manage and live with the disease.
5. Autoimmune disease		Identifying women with autoimmune POI is pertinent, for diagnosing patients with subclinical or latent autoimmune Addison’s disease. Special consideration is needed in cases where the second line of treatment includes chemotherapeutic drugs that are gonadotoxic.	It is important to evaluate the ovarian reserve in women diagnosed with autoimmune disease due to the coexistence of related conditions. In the initial phase of autoimmune POI, ovarian reserve is still normal, allowing cryopreservation of gametes.
6.1. Chemotherapy	When applicable, a treatment combination that is the least gonadotoxic is preferable. Current fertility preservation techniques are available, cryopreserving oocytes, embryos, and OTC when the ovarian tissue is free of residual disease.		Patients can cryopreserve oocytes, embryos, or ovarian tissue after the administration of chemotherapy, particularly in cases where treatments are only mildly damaging.
6.2. Radiation	Oocyte/embryo cryopreservation, OTC, and oophorpexy.		
6.3. Surgical	When possible, the least damaging technique for the ovarian reserve should be used. Gamete banking or OTC are available.		
7. Environmental exposure	Lifestyle modification is a robust mode of primary prevention of POI. Quitting cigarette smoking. In cases of exposure to hazardous chemicals at their workplace, women should be educated about the risk and adequately protected, particularly if they plan to conceive or are already pregnant.		Discontinuing hazardous exposure. Fertility preservation should be considered in cases when there is still a substantial ovarian reserve.
